# Evaluating cost-effectiveness in the management of neuroendocrine neoplasms

**DOI:** 10.1007/s11154-020-09608-y

**Published:** 2020-11-06

**Authors:** B. E. White, R. Mujica-Mota, T. Snowsill, E. M. Gamper, R. Srirajaskanthan, J. K. Ramage

**Affiliations:** 1 Department of Gastroenterology, Basingstoke and North Hampshire Hospital, Hampshire Hospitals NHS Foundation Trust, Hampshire, UK; 2 Kings Health Partners Neuroendocrine Tumour Centre of Excellence, London, UK; 3 Department of Health Economics, University of Leeds, Leeds, UK; 4 Department of Health Economics, University of Exeter, Exeter, UK; 5 Innsbruck Institute of Patient-centered Outcome Research (IIPCOR), Innsbruck, Austria

**Keywords:** Neuroendocrine neoplasia, Health economic evaluation, Neuroendocrine tumour, Carcinoid tumour, Carcinoid syndrome

## Abstract

The rapid evolution of novel, costly therapies for neuroendocrine neoplasia (NEN) warrants formal high-quality cost-effectiveness evaluation. Costs of individual investigations and therapies are high; and examples are presented. We aimed to review the last ten years of standalone health economic evaluations in NEN. Comparing to published standards, EMBASE, Cochrane library, Database of Abstracts of Reviews of Effects (DARE), NHS Economic Evaluation Database and the Health Technology Assessment (HTA) Database were searched for health economic evaluations (HEEs) in NEN published between 2010 and October 2019. Of 12 economic evaluations, 11 considered exclusively pharmacological treatment (3 studies of SSAs, 7 studies of sunitinib, everolimus and/or 177Lu-DOTATATE and 1 study of telotristat ethyl) and 1 compared surgery with intraarterial therapy. 7 studies of pharmacological treatment had placebo or best supportive care as the only comparator. There remains a paucity of economic evaluations in NEN with the majority industry funded. Most HEEs reviewed did not meet published health economic criteria used to assess quality. Lack of cost data collected from patient populations remains a significant factor in HEEs where clinical expert opinion is still often substituted. Further research utilizing high-quality effectiveness data and rigorous applied health economic analysis is needed.

## Introduction

Neuroendocrine neoplasms (NEN) occur throughout the body, the most common sites being pulmonary and digestive. NEN range from well-differentiated neuroendocrine tumours (NET) to poorly-differentiated carcinomas termed neuroendocrine carcinomas (NEC) [2]. They have varying growth characteristics: low grade and indolent to high grade and aggressive. NEN arising in the gastrointestinal tract are termed gastroenteropancreatic neuroendocrine tumours (GEP NETs).

The move from International Classification of Diseases (ICD) 10 to ICD-0-3 resulted in a more accurate classification of NEN. A further update to grade and stage classification in 2018 gave more reliability across users and systems [36]. As a result of these improvements the histological nomenclature and classification of NEN is starting to more accurately reflect the true incidence and prevalence of NEN and its subsets.

NEN incidence is rising [12, 19]. NEN incidence in England is 9.37 per 100,000 according to the UK’s National Cancer Registration and Analysis Service(NCRAS) [62]. Rising incidence of these types of tumour is as yet unexplained but be real, or may relate to increased diagnosis [22] or improved classification systems. In 2017, NEN prevalence in England was 48 per 100,000 [62]. This is greater than most other upper gastrointestinal cancers (e.g. the incidence of gastric cancer stood at 29.6 per 100,000 in 2010) [6]. In the United States, annual age-adjusted incidence of NETs was 1.09 per 100,000 in 1973 and increased to 6.98 per 100,000 by 2012[12]. Increasing incidence of NEN has also been observed in Spain [18], France [33] and Italy[5].

The heterogeneous clinical presentation and biology of NEN with often vague abdominal symptoms can present a challenge in diagnosis and management. Delayed diagnosis, which increases health care costs in a range of diseases [17, 24, 39], is likely to be factor in NEN. Patients with NEN are often initially misdiagnosed or can experience delay to diagnosis of up to five years [4]. High resource utilisation before diagnosis [53] and an expensive diagnostic process [23] contribute to increased overall costs in this phase. An advanced stage at diagnosis confers significantly poorer outcomes of NENs compared with non-NENs at the same anatomical site [19].

Treatment options comprise surgery, targeted therapies, peptide receptor radionuclide therapy (PRRT), trans-arterial chemoembolization (TACE), thermal ablation of liver lesions in the form of radiofrequency ablation (RFA) or microwave ablation (MWA), chemotherapy and liver transplant. The mainstay of treatment in GEP-NETs is long term long-acting somatostatin analogues [60]. Treatment in NEN is also long-term with surgery for advanced GEP-NETs unlikely to be curative [60]. There is a lack of evidence on the best way to investigate and diagnose a NEN in the face of innovative, costly investigation and treatment. Therapy for NEN is reported by patients to vary internationally, and there has been criticism about disparities in access to treatments across countries [35]. A multidisciplinary team approach is important for the optimal treatment of patients with GEP NETs [30, 59].

The total cost of managing illness in NEN is an important figure for healthcare commissioners to achieve an adequate and fair distribution of limited resources across healthcare systems. Some studies have estimated the cost using combinations of physician surveys [8] and registry linkage [23, 34] but results are not necessarily applicable to the UK due to differences in pricing of treatments, health systems and economies. Cost of care in cancer in the UK has been calculated [32]. Decisions about which costs fall within and outside the scope and perspective depend on the decision-maker and can have a significant impact on the resulting cost of illness [25]. In terms of cost-effectiveness, quality-adjusted life years (QALYs) are often the measure of health benefit preferred, and these require appropriate estimation of utility values (QALY weights). The preferred measure of health-related quality of life by the National Institute for Clinical Excellence (NICE) in adults has been EQ-5D since 2008 [42].

Pharmaceutical pricing is the single largest factor impacting the cost of care in the form of long acting somatostatin analogues and targeted therapies [3, 23, 46]. A large registry-linked study found the pharmaceutical cost burden formed 42% of the yearly total cost of managing NEN [34]. In the maintenance phase of illness (more than a year after diagnosis), per patient costs in NEN were found to be triple of those in colon cancer due primarily to pharmaceutical costs [23].

There is a high cost of biochemical testing of blood and urine in NEN due to measurement of serum chromogranins and urine 5-Hydroxyindoleacetic acid. Radiological monitoring post-diagnosis and treatment for NEN is also costly (Table 1). Most patients will need computed tomography (CT) at diagnosis with many additionally needing magnetic resonance imaging (MRI) [16]. In stage 2–3 disease follow up CT can be six monthly for up to five years, but in stage 4 disease radiological monitoring can be for many years longer. Positron emission tomography (PET) scans are now commonly performed at diagnosis with many patients needing fluorodeoxyglucose (FDG-PET) as well as Dotatate-PET to establish “radiological grading” [57].

**Table 1:** Dotatate-PET scans are costed at £1800 in one London centre (Nuclear Medicine Department, King’s College Hospital- personal communication) with an average stage 4 disease patient requiring one or two scans

Imaging modality	Cost (£)
FDG PET	578
Dotatate PET	1800
Tektroyd	1273
Pre SIRT shunt scan (MAA)	963

Selective Internal Radiation Therapy (SIRT) techniques can also be utilized which need a Macroaggregated Albumin (MAA) scan to be done beforehand

### An overview of healthcare costs in England (Table 1) [45]

Somatostatin analogue costs range between £800–£1000 per month (pm). Targeted therapies such as everolimus or sunitinib cost £2000–3000 pm [45]. PRRT costs £40–50,000 per patient with many patients now having multiple cycles to control disease burden (Kings Health Partners Business Intelligence unit, personal communication). Chemotherapy costs are mostly due to high-cost drugs, with chemotherapy unit attendance contributing a much smaller part [45].

The most frequently used chemotherapy regimens are Temozolamide with Capecitabine oral combination therapy or single agent platinum-based intravenous treatment. In the treatment of grade 2 metastatic NEN, temozolamide is estimated to cost £1176 per 5 day cycle and capecitabine £120 per 14 day cycle [43]. For grade 3 NEN, a platinum-based intravenous regimen would cost £76.18 per cycle for carboplatin and £30.89 for etoposide [44], in addition to the attendance cost estimated at £130 per day on a chemotherapy unit [45].

Reimbursed costs for surgery and interventional radiology treatments in England (Table 2) [45] HRG = Healthcare Resource Group

**Table 2:** National Health Service healthcare resource group (HRG) codes and tariff (in GB Pounds) covering the whole cost of each admission for the procedure

Procedure	HRG	Tariff (£)
Orthotopic transplantation of whole liver	GA15	80,000
Right hemihepatectomy NEC	GA04	10,801
Pancreaticoduodenectomy NEC	GA03	9263
Left pancreatectomy NEC	GA05	8405
Right hemicolectomy	FF32	5947
Small bowel resection	FF21	6109
RFA to liver	GA13	2141
Thermal ablation of single lesion of liver	GA13	2141

Health economic evaluations (HEEs) are increasingly adopted by the UK government to assess treatments [49]. HEEs have become an integral part of rational decision making in health care and form a key part of health technology assessments (HTAs). NICE requires for all new pharmaceuticals to have HEE and will often commission HEEs where there is an absence of existing cost-effectiveness evidence. HEEs appear to have a major influence in NICE’s decision process; one research paper demonstrated the economic calculation could predict 82% of NICE decisions [11].

In HEE the following questions are posed: firstly whether a treatment is worth doing compared with other things we could do with the same resources, and secondly if these resources should be spent in this way and not on something else [14] .

### Types of health economic evaluation (Table 3)

**Table 3:** The ICER or incremental cost-effectiveness ratio is the amount of extra cost which will be incurred for a unit gain of health benefit

Health economic evaluation	Description
Cost-minimisation analysis (CMA)	Assumes there are no differences in the benefits so only costs are considered
Cost-consequence analysis (CCA)	Typically identifies multiple benefits and presents these alongside costs
Cost-effectiveness analysis (CEA)	Identifies a single benefit measure and reports the incremental cost-effectiveness ratio (ICER)
Cost-utility analysis (CUA)	A special case of CEA where the benefit is a composite of health-related quality and quantity of life utility measure e.g., quality-adjusted life years (QALYs)
Cost-benefit analysis (CBA)	Identifies one or more benefits and assigns monetary values to these benefits to be offset against costs.

A QALY or quality-adjusted life year is equal to 1 year of life in perfect health. QALYs can be calculated by estimating the years of life remaining for a patient following a particular treatment or intervention and weighting each year with a quality-of-life score (on a 0 to 1 scale)

## Aim

The aim of the paper was to systematically review and critically evaluate standalone English-language literature on health economics in NEN over the last decade. Previous reviews [9, 21] found limited numbers of health economic evaluations. Novel therapeutics also justify a focus on more recent evaluations.

## Methods

**Fig. 1:**
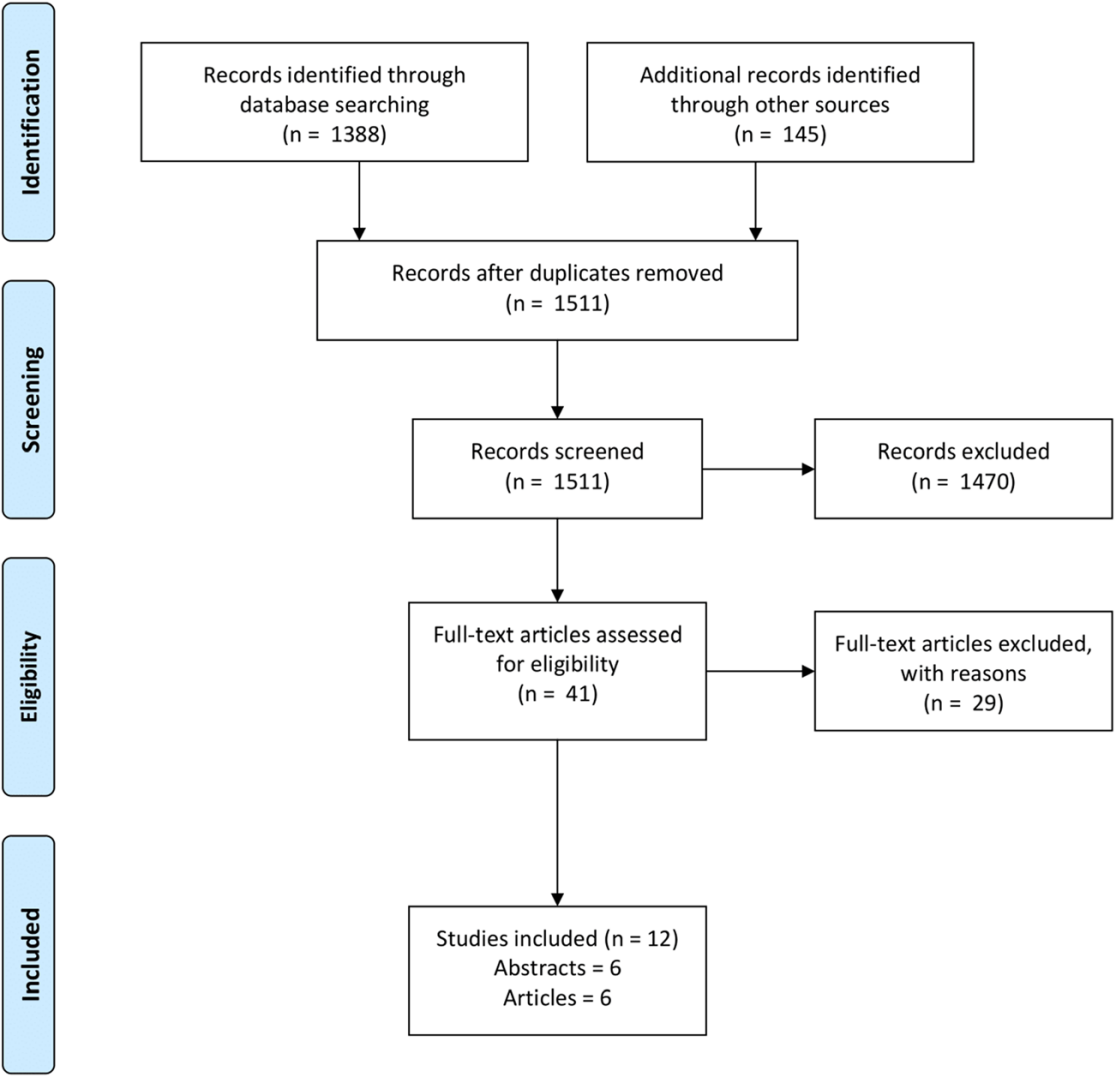
PRISMA flow chart of search strategy

A literature search was performed including EMBASE, Cochrane library, Database of Abstracts of Reviews of Effects (DARE), NHS Economic Evaluation Database and the Health Technology Assessment (HTA) Database. The last three were searched using the Centre for Reviews and Dissemination (CRD) Database. A Preferred Reporting Items for Systematic Reviews and Meta-Analyses (PRISMA) flow diagram was generated. (Fig. 1).

Articles were searched by citation and abstract. Papers published from 2010 to October 2019 in English were included. We looked at treatment search terms, taking into account terms used in previous reviews by Chau et al. [9] and Grande et al. [21] (Table 4). See Appendix 1 for full search strategy terms. Further literature, including grey literature, was identified using a Google scholar search using the same terms. Citation searching was performed to ensure completeness.

**Table 4:** Search terms

Neuroendocrine tumour terms	Treatment terms	Health economics terms	Study type terms
Neuroendocrine Tumo* Neuroendocrine Neoplasia Carcinoid tumo(u)r/syndrome	Octreotide Angiopeptin Alpha interferon (& similes) Everolimus Rapamycin Sunitinib Lutetium 177 (& similes) Lanreotide Sirolimus Sunitinib or 177lu) Chemotherapy Radiotherapy Ablation Radioemboliz(s)ation Artificial emboliz(s)ation Surgery Trans arterial chemoemboliz(s)ation Telotristat	Economics Hospitalization & Hospitalization costs (& similes) Cost of illness Mortality Ambulatory care Work Disability Health care cost (& similes) Treatment pattern Drug utilization (& similes) Unmet needs Quality of life Health economic analysis Health economics Health care utilization Resource utilisation (& similes) Financial toxicity Cost-effectiveness Cost of disease/sickness/illness Burden of disease/illness Hospital/illness/sickness/health*/medical care cost Drug cost Cost of medical care/drug/healthcare (& similes)/treatment(& similes) Economic burden Economic impact Health services Drug prescriptions Direct cost Intangible cost Surg* cost Payment Pharmacoeconomic Absenteeism Illness/sick day Sick leave Work absen* Retirement Work/work day/time loss Work incapability/ incapacity Workers compensation	Registries Observational studies Review articles Guidelines or consensus pieces Meta-analyses Systematic reviews Surveys

The following were excluded: papers which were not economic evaluations or reviews of economic evaluations, papers which did not relate to NEN, papers not in English and budget impact studies. Cost of illness studies were excluded since limiting analysis strictly to HEEs was considered optimal.

Initial screening for excluding the above categories was conducted by a researcher. Subsequently, each paper was reviewed by three experts. The reviewers agreed on the final group of articles to be included in the review. If reviewers disagreed, a majority decision was taken.

Data on the methods and results of studies were abstracted into a standardised table by a researcher (BW) and checked by an experienced health economist (TS). Studies were critically appraised by TS using the CHEC list for trial-based economic evaluations and the Philips checklist for model-based economic evaluations [15, 47]. Results of data extraction were analysed by all authors.

## Results

EMBASE, Cochrane and CRD search yielded 1388 articles. Google Scholar and citation tracking combined gave 145 results, making a total of 1533 articles. 22 articles were duplicates leaving 1511. 1470 articles were excluded. 41 articles were retrieved of which 29 were excluded making 12 total. There were 6 papers and 6 abstracts included.

Table 5a and b display the country of origin and funding source for the studies. Table 6 displays the study characteristics and Table 7 study results.

**Table 5:** Countries of origin and funding sources

a Country of origin	
Country	Number
United States	4
Brazil	1
Canada	1
France	1
Netherlands	1
Poland	1
Portugal	1
Scotland & Wales	1
United Kingdom	1
Total	12
b Funding source	
Source	Number
Novartis	2
IPSEN	2
Not stated	5
Lexicon	1
Publicly funded	1
Pfizer	1
Total	12

**Table 6:** Study characteristics

Author & year	Publication type	Patient population & Country setting	Treatments evaluated	Study type: CMA/CCA/CEA/CUA/CBA	Analytical framework (Evaluation with RCT, Observational, Model [DT/Markov /DES])	Main health states/events analysed	Measure of benefit (discount rate)	Costs measured (currency, price year, discount rate)	Time horizon	Source of effectiveness data	Funding source
Takemoto 2010 [58]	Abstract	Metastatic midgut NET. Brazil	Octreotide-LAR vs. BSC	CEA	Markov^a^	PFS, Progression, Death	PFYG (5%)	N/s (BRL, N/s, 5%)	10 year	Phase III PROMID trial [52]	N/s; Two co-authors employed by Novartis
Soares2011 [54]	Abstract	Metastatic pancreatic NET. Portugal	SU + BSC vs. Placebo+BSC	CEA	Markov	PFS, Post-progression, Death	LY gain (5%), adverse events	N/s (EUR, N/s, 5%)	Lifetime	A6181111 [51]	N/s; Two co-authors employed by Pfizer
Walczak 2012 [61]	Abstract	Advanced & Metastatic pancreatic NET. Poland	SU + BSC vs. Placebo+BSC	CUA, CEA	Markov	N/s but expected to be PFS, progression and death	QALY, LYG, PFYG (all 3.5%)	Drug acquisition and administration, diagnostic and monitoring, SSA, BSC, severe AEs, palliative care (PLN/EUR, 2011, 5%)	Lifetime	A6181111 [51]	N/s; Two co-authors employed by Pfizer
Marty 2012 [37]	Paper	GEP-NETs (also acromegaly). France	Somatuline Autogel® Vs. Sandostatin LAR	CMA	Decision Tree	Successful injection vs clogging	None (N/A)	Drug acquisition and administration (EUR, 2010, N/A)	n/a	Adelman et al. [1]	IPSEN
Johns 2012 [26]	Abstract	Advanced metastatic well-differentiated pancreatic NET. Scotland & Wales	SU + BSC vs. Placebo+BSC	CUA	Markov	PFS, Post-progression, Death	QALY (3.5%)	N/s (GBP, N/s, 3.5%	N/s	HTA submissions to Scotland and Wales (Jan and Mar 2011), Resource use estimated by two UK pNET experts, and PROMID [52].	Pfizer
Kansal [28]	Abstract	Advanced metastatic well-differentiated pancreatic NET. Netherlands	SU + BSC vs. Placebo+BSC	CUA/CEA	Markov	PFS, Post-progression, Death	LYG, QALY (all 1.5%)	N/s (EUR, N/s, 4%)	Lifetime	Transition probabilities and AE rates from PROMID [52]	Pfizer
Casciano 2012 [7]	Paper	Advanced moderate to well-differentiated NET of pancreas. US	EVO vs SU	CUA/CEA	Partitioned survival	Stable disease with no AEs, stable disease with AEs, Disease progres- sion, Death	LYG, QALY (all 3%)	Drug acquisition, symptomatic care, procedures/tests, physician visits, hospitalisations, adverse events, post-progression costs, end-of-life care (USD, N/s, 3%)	Lifetime (20 year)	MAIC of RADIANT-3 [65] versus A6181111 [51]	Novartis
Spolverato 2015 [55]	Paper	NET liver metastases. US	Hepatic resection vs Intraarterial therapy	CUA/CEA	Markov	Alive post-HR, Alive post-IAT, Dead	QALY (3%)	Hepatic resection surgery, intraarterial therapy, systemic therapy (USD, N/s, 3%)	Lifetime	Mayo, de Jong et al. [38]	N/s
Chua 2018 [10]	Paper	Advanced or metastatic NET of GI or lung origin	EVO + BSC vs BSC	CUA	Partitioned survival	Stable disease, disease progression, and death	LYG, QALY (all 5%)	Drug acquisition, physician visits, procedures, tests, adverse events (CAD, 2015, 5%)	10 years (30 years in sensitivity analysis)	RADIANT-4 [66]	Novartis
Joish 2018 [27]	Paper	US, patients with carcinoid syndrome diarrhoea whose symptoms remain uncontrolled with SSA alone	Telotristat + SSA LAR vs. SSA LAR	CUA	Markov	Adequate Control; Control Not Adequate; Dead	QALYs (3%)	Outpatient visits, pharmacy, ED visit, inpatient stay, other medical encounters, drug acquisitions (USD, N/s, 3%)	Lifetime	12-week TELESTAR responder analysis [29]	Lexicon
Mujica-Mota 2018 [41]	Paper	UK, advanced, unresectable or metastatic neuroendocrine tumours with disease progression	Everolimus, Sunitinib and 177Lu-DOTATATE	CUA	Partitioned survival	Pre-progression; Post-progression; Death	PFY, LY (0%); QALY (3.5%)	Drug acquisition and administration, medical management, disease monitoring, severe AEs, end-of-life care (GBP, 2016, 3.5%)	Lifetime (40 years)	RADIANT-3 [65], A6181111 [51], RADIANT-4 [66], NETTER-1 [56]	NIHR
Ray 2019 [50]	Abstract	US. Unresectable, well-differentiated, advanced GEP-NETs	OCT escalation vs. OCT + PRRT vs. OCT + liver directed therapy vs. everolimus vs. LAN	CMA	Decision tree model	None	None	Drug, administration, adverse event, patient monitoring costs (USD, N/s, N/s)	6 month	N/s	Ipsen

Identified as Markov model, but insufficient evidence provided to confirm not a partitioned survival model

Key: CEA, cost-effectiveness analysis; CMA, cost-minimisation analysis; CUA, cost-utility analysis; LY(G), life years (gained); HTA, Health Technology Assessment; MAIC, Matching adjusted indirect comparison; PFY(G), progression-free years (gained); QALY, quality-adjusted life years; CE, cost-effectiveness; DT, decision tree; DES, discrete event simulation; RCT, randomized controlled trial; OCT, Octreotide; LAN, Lanreotide; SU, Sunitinib; EVO, Everolimus; AE, Adverse events; pNET, pancreatic neuroendocrine tumour; N/s, Not stated

**Table 7:** Study results

Author & year	Treatments	Patient characteristics	PFS life years	Life Years	Discounted QALYs (unless stated otherwise)	Discounted costs	ICER	Key uncertain factors (e.g. from sensitivity analysis)	Comments (may highlight authors’ conclusions/caveats)
Takemoto 2010 [58]	Octreotide LAR vs. BSC	Metastatic midgut NET	1.07 v 0.46 (+0.6)	N/s	N/s	303,111 v 275,497	~BRL46,000/PFYG^a^	Resource use partly estimated by clinical experts. No OS.	OCT-LA gives longer time to progression compared to BSC in metastatic midgut NET, Brazil, private setting
Soares 2011 [54]	SU + BSC vs. Placebo+BSC	Well-differentiated advanced or metastatic pNET	N/s	+1.83	N/s	54,215 v 10,239	€24,035/LY (RPSFT); €34,387/LY (ITT)	RPSFT method limitations	SU cost effective compared to BSC in pNET, in Portuguese NHS
Walczak 2012 [61]	SU + BSC vs. Placebo+BSC	Well-differentiated unresectable or metastatic pNET	N/s	N/s	N/s	N/s	[EUR; PNHF, PNHF+patient] 20,441/QALY, 20,461/QALY 14,188/LYG, 14,201/LYG 19,386/PFYG, 19,405/PFYG	N/s	SU in combination with BSC prolongs OS and time to next progression
Marty 2012 [37]	Somatuline Autogel® Vs. Sandostatin LAR	GEP-NET Assumed 50% in hospital and 50% in community	N/A	N/A	N/A	[Cost per successful injection] France: €1305 v €1340 (−€34.90) Germany: €2322 v €2413 (−€91.10) UK: €875 v €1018 (−€142.90)	N/A	Only direct costs included, admin costs used average nurses wage	Only considers costs of drug acquisition and nurse time for administration. Assumes clinical equivalence.
Johns 2012 [26]	SU + BSC vs. Placebo+BSC	Well-differentiated advanced or metastatic pNET	N/s	N/s	+1.39	£41,803 v. £10,387	£22,587/QALY	Resource use was estimated by two UK pNET experts. Extension-phase data included for overall survival (OS)	SU treatment for pNET is cost-effective by accepted UK threshold for end-of-life treatments, but data not presented to support eligibility for this threshold.
Kansal 2012 [28]	SU + BSC vs. Placebo+BSC	Well-differentiated advanced or metastatic pNET	N/s	+1.32	+0.78	€48,388 v €7267	€31,067/life year gained and €52,401/QALY	Resource use for BSC and AE management was estimated by Dutch clinical experts	SU showed improved effectiveness, and cost-effective ICER
Casciano 2012 [7]	EVO vs SU	Advanced, progressive pNET	1.20 v 1.04 (+0.153)	3.30 v 2.85 (+0.448)	2.19 v 1.82 (+0.304)	$194,053 v $181,381 (+$12,673)	$28,281/LYG $41,702/QALY	Relative effectiveness (PFS and OS) of EVO v SU imprecisely estimated by indirect comparison	Analysis limited by reliance on indirect comparison of two phase III studies. Probability EVO cost-effective is 53% if CE threshold is $50,000/QALY, 69% if CE threshold is $100,000/QALY
Spolverato 2015 [55]	Hepatic resection vs. Intra Arterial therapy (for liver metastasis)	57-year-old male patient with metachronous symptomatic NELM that involved <25% of the liver in the absence of extrahepatic disease; also - 57yo >25% and - >65yo with >25%	N/s	N/s	5.92 v 3.92 (+2.00)^b^	$84,535 v $67,689 (+$16,855)^c^	$8427/QALY for HR	Inability to include quality-adjusted life expectancy owing to the lack of data on the estimated utilities of different health states among patients with NELM	With low disease burden, HR should be considered as first-line. But among asymptomatic patients with extensive liver disease, IAT has similar survival and is more cost effective
Chua 2018 [10]	EVO + BSC vs BSC	Advanced or metastatic GI or lung NET	1.27 v 0.90 (+0.373)	3.85 v 3.02 (+0.823)	2.86 v 2.24 (+0.616)	CA$146,137 v CA$56,342 (+CA$89,795)	CA$109,116/LYG CA$145,670/QALY	Immature evidence for overall survival, uncertainty regarding dose intensity, time horizon very important	At WTP threshold of CA$150,000/QALY everolimus has 52% probability of being cost-effective
Joish 2018 [27]	Telotristat + SSA vs. SSA alone	Carcinoid syndrome diarrhoea	N/s		2.33 v 1.67 (+0.66)	$590,087 v $495,125 (+$94,962)	$142,545/QALY	Probability of death in this cohort was calculated using u5-HIAA as a predictor of mortality based on an analysis in patients with mid-gut carcinoid tumours. Utility estimates for active disease and remission states were derived from similar symptoms in Ulcerative Colitis.	A WTP threshold of $150,000 per QALY gained was used as a benchmark for cost-effectiveness, based on the most recent ICER Value Assessment Framework cost-effectiveness threshold range
Mujica-Mota 2018 [41]	Sunitinib v Everolimus v vs. BSC	Pancreatic NET	1.60 v 1.28 v 0.57	6.39 v 4.69 v 3.46	+1.32 v + 0.59 v [ref]	£43,192 v £42,646 v £15,761	Sunitinib v BSC: £20,717/QALY; Everolimus extendedly dominated	Indirect comparisons of RCTs have been used when there is evidence that the populations are imbalanced for prognostic factors and may be imbalanced for effect modifiers.	Given NICE stated cost-effectiveness threshold of £20,000–30,000/QALY, based on list prices, only sunitinib considered good value for money (in England and Wales).
	Everolimus v BSC	GI and lung NET	1.42 v 0.83	6.21 v 4.82	3.74 v 3.05	£47,334 v £16,526	£44,557/QALY		
	Everolimus v BSC	GI (midgut) NET	2.08 v 1.44	7.50 v 7.05	4.37 v 4.19	£55,842 v £21,119	£199,233/QALY		
	^177^Lu-DOTATATE v Everolimus v BSC	GI (midgut) NET	5.41 v 2.07 v 1.43	6.66 v 5.75 v 4.90	4.19 v 3.57 v 3.11	£83,667 v £52,018 v £16,628	^177^Lu-DOTATATE v BSC: £62,158/QALY; Everolimus extendedly dominated		
Ray 2019 [50]	OCT escalation vs. OCT + PRRT vs. OCT + liver directed therapy vs. everolimus vs. LAN	GEP-NET previously treated with octreotide monotherapy	NA	NA	NA	LAN: $44,462 OCT 30 mg Q3W: $52,873 OCT 40 mg Q4W: $53,041 OCT + LDT: $72,152 Others: N/s	NA		LAN was the lowest cost treatment option over a 6-month interval for GEP-NET patients who progressed on OCT, but clinical effectiveness has not considered and clinical appropriateness must be considered when transitioning patients

^a^The authors report an incremental cost per progression-free year gained of 28,706 BRL, but this is inconsistent with the total costs reported and cannot be derived from other figures presented

^b^In Table III of Spolverato et al. these figures are the other way around, but it is believed that this is the true ordering

The Philips checklist [47] was applied to all economic evaluations (Appendix 2). All studies clearly stated their decision problem and most studies clearly reported the scope and perspective of their study, including the time horizon. Studies generally did not present evidence for the structures of their models, although most studies adopted similar structures (health states for pre-progression, post-progression and death), which likely arises as a result of the prominence of progression-free survival as a key endpoint in RCTs.

A number of studies excluded relevant comparators without justification (e.g., studies evaluating sunitinib but not everolimus). Most studies did not describe their methods for identifying data to inform model inputs and it was often unclear how relevant the utilities or QALY weights incorporated within models were. Exploration of uncertainty was sporadic, with parameter uncertainty most likely to be explored (through one-way sensitivity analyses and probabilistic sensitivity analysis), and most studies did not report internal and external validation.

Results from economic evaluations are grouped by comparison of treatments. Of twelve economic evaluations, eleven considered exclusively pharmacological treatment (three studies of SSAs, seven studies of sunitinib, everolimus and/or 177Lu-DOTATATE, one study of telotristat ethyl) and one compared surgery with intraarterial therapy. Seven studies of pharmacological treatment had placebo or best supportive care as the only comparator.

## Somatostatin analogues (SSAs)

Marty et al. [37] developed a decision tree model to perform a cost-minimisation analysis of lanreotide (extended release formulation; trade name Somatuline Autogel® or Somatuline Depot®) versus octreotide (trade name Sandostatin LAR®). The measure of benefit was a successful injection (as there is a risk of clogging), and costs were estimated from French, German and UK healthcare payer perspectives and reported in 2010 Euros. The study found, through a combination of longer administration times and higher risk of clogging with octreotide, lanreotide to be cheaper per successful injection (France €34.90, Germany €91.10, UK €142.90). The study included only drug acquisition and administration costs, and did not include costs of adverse events or any measure of health benefit. The data source for administration time was a study in which nurses were timed performing injections into pads. The nurses were shown an instructional video on injection preparation and administration for lanreotide prior to administering lanreotide, whereas they were only provided with printed instructions for octreotide. Prior to this they were also given a demonstration and explanation of the features of the lanreotide pre-filled syringe and were asked to describe its most important characteristics.

Takemoto et al. [58] developed a three health state Markov model to assess the costs and consequences of octreotide versus best supportive care (BSC) in patients with metastatic midgut NET. The measure of benefit was progression-free survival. Costs were estimated from the private payer perspective and reported in Brazilian real (BRL). The primary source for effectiveness data was published data from the phase III PROMID trial [52]. Subjects remained on treatment until progression and resource use was estimated through published data and input from clinical experts. They state that octreotide is a clinically effective option to control tumour growth in patients with metastatic midgut NET. The authors state that since PROMID was not designed to evaluate OS they were unable to calculate life years gained.

Ray et al. [50] developed a decision tree model to assess the cost of treating unresectable, well-differentiated, advanced GEP-NET patients over a 6-month time horizon after they progress on octreotide. The stated basis is evidence of benefit to patients from switching to lanreotide after octreotide prior to modifying treatment class. There was no measure of health benefit (i.e., cost-minimisation analysis). Drug acquisition/administration, serious adverse event and patient management costs were considered. Patients could utilize octreotide escalation (30 mg every 3 weeks or 40 or 60 mg every 4 weeks), OCT plus PRRT, OCT plus liver-directed therapy, everolimus or lanreotide every 4 weeks. Costs were estimated from a US insurance payer perspective and reported in US dollars. Results were that lanreotide was found to be cost-saving versus alternatives when used post-octreotide. The authors state that clinical appropriateness must be considered when transitioning patients.

## Everolimus, sunitinib and ^177^Lu-DOTATATE

Casciano et al. [7] developed a partitioned survival model to conduct a cost-utility analysis of sunitinib versus everolimus in patients with advanced, progressive pancreatic NET from a US payer perspective. The measure of benefit was QALYs and cost-effectiveness thresholds of $50,000 and $100,000/QALY were considered. Data from two RCTs (A6181111, RADIANT-3) were synthesised using the matching-adjusted indirect comparison method and using individual patient data from RADIANT-3[51, 65]. For PFS this was an anchored comparison (with placebo control as a common comparator), but for OS this was an unanchored comparison, because significant treatment switching was observed after disease progression in the control arms, leading to confounding of OS estimates. The study estimated that everolimus would improve life expectancy and QALYs at an additional cost, with an ICER of $41,702/QALY. The primary threat to the validity of this study is the use of an unanchored indirect comparison of OS from two studies, which requires assumptions which are acknowledged to be generally very difficult to justify [48].

Chua et al. [10] developed a partitioned survival model to conduct a cost-utility analysis of everolimus (with BSC) versus BSC alone in patients with advanced or metastatic NET of GI or lung origin from a Canadian healthcare payer perspective (although described by the authors as a societal perspective). The measure of benefit was QALYs and a cost-effectiveness threshold of CA$150,000 was considered. Intention to treat data from RADIANT-4 [66] informed PFS and OS estimates up to month 26, however after this point a proportional hazards assumption for OS was imposed. Health state utility values were estimated by mapping from FACT-G measured in RADIANT-4 to EQ-5D values. The study estimated that everolimus would improve life expectancy and QALYs at an additional cost, with an ICER of CA$145,670/QALY. The ICER was sensitive to the hazard ratio for long-term OS and to the time horizon, suggesting that the economic value is derived in significant part from extrapolation beyond the trial evidence.

Mujica-Mota et al. [41] developed a partitioned survival model to conduct a cost-utility analysis of sunitinib, everolimus, and ^177^Lu-DOTATATE versus BSC in patients with advanced unresectable or metastatic NET. The measure of benefit was QALYs and costs were included from an NHS and personal social services perspective. Four RCTs [51, 56, 65, 66] were used to estimate PFS and OS, however these RCTs had heterogeneous patient populations. Sunitinib could only be included when the population was limited to pancreatic NET, reflecting its licensed indication, and ^177^Lu-DOTATATE could only be included when the population was limited to gastrointestinal (midgut) NET. Ultimately three versions of the model were constructed: for pancreatic NET data from RADIANT-3 [65] and A6181111 [51] were combined; for GI (midgut) NET data from RADIANT-4 [65] and NETTER-1 [56] were combined; and for GI and lung NET only data from RADIANT-4 was included. The study found that everolimus was unlikely to be cost-effective in any of the settings at its list price, sunitinib was likely to be cost-effective in pancreatic NET, and ^177^Lu-DOTATATE was unlikely to be cost-effective in GI (midgut) NET. The main limitation of the study is that indirect comparison was required for evidence synthesis because all RCTs were placebo-controlled rather than being head-to-head RCTs of active treatments.

Soares et al. [54], Walczak et al. [61], Johns et al. [26] and Kansal et al. [28] developed models to conduct cost-utility and cost-effectiveness analyses of sunitinib plus BSC versus placebo plus BSC in advanced unresectable or metastatic pancreatic NET. All four studies were supported by Pfizer. The measure of benefit was life years (LY) in Soares et al. [54] but was QALYs in the other study abstracts. The cost-effectiveness threshold was not stated in Soares et al., but anecdotally Portugal adopts a threshold of €30,000/QALY [67]. The cost-effectiveness threshold was stated by Walczak et al. [61] to be 99,543 PLN/QALY in Poland, and was stated by Johns et al. [26] to be £50,000/QALY in the UK for end-of-life treatments. Kansal et al. [28] did not state a cost-effectiveness threshold. A single RCT (A6181111) was used as the source of evidence in all studies. In Soares et al. [54] and Johns et al. [26] OS was adjusted for treatment switching using the rank-preserving structural failure time (RPSFT) method.

Soares et al. [54] estimated a LY gain of 1.83 with an ICER of €24,035/LY. When using ITT analysis (instead of RPSFT) the ICER increased to €34,387/LY. Walczak et al. [61] did not report costs and benefits separately but reported an ICER of 84,214 PLN/QALY (€20,441/QALY). Johns et al. [26] estimated an ICER of £22,587/QALY. Kansal et al. [28] estimated an ICER of €52,401/QALY.

## Telotristat ethyl

Joish et al. [27] developed a Markov model to conduct a cost-utility analysis of telostristat ethyl added to octreotide (TE + SSA) versus octreotide alone (SSA) in patients with carcinoid syndrome diarrhoea (CSD). Costs were included from a third-party US payer perspective and the measure of benefits was QALYs. The cost-effectiveness threshold was $150,000/QALY but thresholds of $300,000 and $450,000/QALY were also argued to be relevant as CSD was argued to be an ultra-orphan condition. The model included states for adequate control and inadequate control, as well as death. Patients in the SSA arm could transition from adequate control to inadequate control but patients in the TE + SSA arm could not. The key source of effectiveness data was a multi-country RCT [29]. Utilities were estimated from a vignette study of ulcerative colitis patients. A biomarker, u5-HIAA, was used as a surrogate outcome which was assumed to mediate a mortality benefit. The study found that TE + SSA increased QALYs by 0.66 at an additional cost of $94,962 (ICER $142,545/QALY). This was considered cost-effective at the thresholds considered but these thresholds are higher than typically used in US economic evaluations ($50,000 and $100,000/QALY).

The most significant threats to validity of results generated from in this case was the assumption that ulcerative colitis health state utility values elicited using vignettes are an appropriate proxy for CSD health states and the surrogate assumption that the observed association between u5-HIAA and mortality is a suitable basis for estimating the effect on mortality of an intervention from its effect on u5-HIAA. It would instead seem more appropriate to take existing measures of health-related quality of life in this disease cohort, e.g., EORTC QLQ-C30 in the TELESTAR study [29], and using a utility mapping algorithm [64] to estimate preference-based utility values. This would narrow the gap in utility between adequate and inadequate control patients.

## Surgery and intra-arterial therapy

Spolverato et al. [55] developed a Markov model to conduct a cost-utility analysis of hepatic resection (HR) versus intraarterial therapy (IAT) for patients with NELM. Costs were included from a US health care provider’s perspective and the measure of benefits was QALYs. The cost-effectiveness threshold was $50,000 per QALY. The only health events incorporated in the model were retreatment and death. The key sources of effectiveness data was a retrospective cohort study [38] of individuals undergoing treatment for NELM by HR or IAT over a 25 year period at one of nine US institutions. In the base case (57-year-old man with metachronous symptomatic NELM involving <25% of liver and no extrahepatic disease) HR was cost-effective versus IAT, however other cases were identified where HR was not cost-effective, e.g., when asymptomatic but with hepatic involvement ≥25%. There was no accounting for confounding in the primary evidence source (since IAT recipients were more likely to have greater hepatic involvement and presence of extrahepatic metastases), so the economic evaluation likely overestimates the survival benefit from HR versus IAT and its cost-effectiveness.

## Discussion

It was stated in the review by Chau et al. [9] that “Although the published literature in the area of NET is substantial, there is a lack of treatment-specific and comparative economic and outcomes research data associated with commonly used treatments”. In a subsequent review in 2019 Grande et al. [21] suggested “further economic evaluations are required to inform healthcare decision-making”. Our review demonstrates health economic literature in NEN which fulfils quality criteria for HEEs is still scarce.

Despite there being more HEE in the literature than in the previous decade there remains a paucity of economic evaluations, the majority of which are partly or wholly industry funded. The lack of cost data collected from relevant patient population remains the main weakness of existing evidence from HEEs. Another limitation relates to the lack of data on medium to long term survival outcomes and therefore the need to rely on clinical expert opinion for predicting those outcomes beyond one or two years after the start of targeted therapies, particularly in gastrointestinal NEN [41].

The long-term treatment of NEN is costly due in the most part to pharmaceuticals. In one cost analysis, the long term follow-up of NEN was significantly more costly when compared to colon cancer. The authors demonstrated that almost all of this increased cost was due to maintaining drug treatments such as SSAs [23]. Due to heterogeneity of treatment pathways in NEN it is difficult to calculate accurate continuing costs for the whole cohort. We would expect further literature to emerge on the cost-effectiveness of lutetium treatment.

A problem frequently observed in RCTs of anti-cancer therapies is that patients in the control arm are allowed to switch to the study drug following disease progression [31]. This leads to confounding in post-progression endpoints, for example overall survival, and renders estimates of these endpoints unsuitable for inclusion in economic models without some form of adjustment. The rank-preserving structural failure time (RPSFT) method was used in three included studies [26, 41, 54] to adjust for this confounding, but this method typically assumes that the treatment effect received by switchers must be the same as the treatment effect in those initially randomised to the study drug, which is unlikely to be true when the main cause for switching is disease progression. Alternative methods for adjusting for treatment switching (such as the inverse probability of censoring weights, IPCW, method), as well as intention-to-treat analyses should be conducted and presented. Soares et al. [54] have demonstrated that these methodological choices can have a very significant impact on cost-effectiveness estimates and therefore lead to decision uncertainty.

Another problem encountered when comparing multiple novel therapies is that there are frequently no head-to-head comparisons of the treatments in randomised controlled trials. Where trials have shared a common comparator (often placebo) indirect comparisons and multiple treatment comparisons [13] can be appropriate under certain assumptions. One of the assumptions is that treatment effect modifiers (patient characteristics which affect the relative effectiveness of one or more treatments, as opposed to simply being prognostic) are distributed equally across studies. This assumption can be relaxed by using population-adjusted indirect comparisons when there is access to individual patient data (IPD) in one of the studies in the indirect comparison [48]. One such approach (matching adjusted indirect comparison, MAIC) weights patients in the trials where IPD are available to match the patient characteristics in another trial before conducting an indirect comparison.

If the aim is to compare across conditions, health-related quality of life in a clinical setting can be measured using a generic preference-based multi-attribute utility instrument, such as the EQ-5D, and should be valued using an appropriate tariff (e.g., from a time trade-off or standard gamble study). However disease-specific instruments are often more sensitive and responsive for certain health states [63]. Areas where inaccuracy was introduced in the economic evaluations reviewed included making certain assumptions without justification [55], using other disease areas as proxies [55], use of vignette studies which value hypothetical disease states [27] and referencing unpublished data [27].

Little is known about preference based values associated with health related quality of life outcomes of targeted therapies after disease progression. Almost all evidence originates from randomised clinical trials which measure these outcomes at fixed time points driven by dosing schedules, which vary across treatments, typically with high drop-out rates. This suggests longitudinal preference based assessment of quality of life outcomes of NEN, based on representative patient cohorts, is needed. This evidence would help distinguish patient preferences and relative effectiveness between targeted therapies in advanced or metastatic pancreatic NETs which have similar clinical outcomes but different safety profiles [41].

All the included studies were model-based economic evaluations rather than being economic evaluations alongside clinical trials (EEACT). EEACT include direct measurements of healthcare resource use, for example through resource use questionnaires, claims and other databases and also measurements of effectiveness, e.g. preference-based health-related quality of life. Model-based economic evaluations can accurately estimate costs in the delivery setting (although this is dependent on having high quality data sources), but are less likely to accurately estimate any knock-on effects on costs [20]. In a single centre study the data collection for an EEACT is typically not prohibitive, but for multi-centre (and particularly multinational) trials, data may be more challenging to collect.

Where studies do not incorporate quality of life (i.e., by using LYs instead of QALYs), decision-makers should be aware that incremental QALYs can be considerably lower than incremental LYs in economic evaluations of cancer treatments unless substantial improvements to quality of life are realised. This means ICERs with QALYs as denominators can be much greater than ICERs with LYs as denominator. Soares et al. [54] estimated an ICER of €24,000/LY, however the best estimate of a cost-effectiveness threshold for Portugal is €30,000/QALY. Assuming a fairly typical utility value of 0.7 this would lead to an estimated ICER of around €34,000/QALY.

The limitations of the study mainly relate to the limitations of our systematic review and also to the available evidence. The search terms may not capture all available literature however to attempt to remedy this citation searching and ‘grey’ literature searches using google scholar were performed. We did not look for articles outside of the English language which meant at least one relevant economic evaluation in NEN was not included which has appeared in a previous review [40]. Although EMBASE and other related databases were included in the search, we did not have access to the DIMDI Superbase, which was used in the Chau et al. [9] review. We did not perform a formal risk of bias quality assessment, rank evidence according to grade or examine publication bias.

Limited data on costs hampers adoption decisions regarding targeted treatments. A salient example is the uncertainty in the value for money of PRRT in progressive or metastatic NET, for which there is strong evidence of clinical benefits in terms of progression free survival, but no data on impact on healthcare resource utilisation and costs [41]. Further research could aim to enrich the evidence base on resource use and costs of NEN using electronic medical records and registry data. Studies using models such as that of Laudicella et al. [32] may generate the required generalizable evidence to inform timely policy decision making.

We recommend that investigators for future trials in NEN should ensure that key endpoints for economic evaluations (progression-free survival and overall survival) are not confounded by crossover (treatment switching), or that if crossover is allowed then studies should make this clear beforehand, and collect all necessary data to support methods for adjusting for crossover with different underlying assumptions (e.g., IPCW and RPSFT or iterative parameter estimation methods). The results of each of these different methods should be presented alongside intention-to-treat analyses. Investigators should release anonymised patient-level data to support more reliable syntheses of studies and ideally collect preference-based health-related quality of life measures (e.g., EQ-5D) on a schedule which minimises bias (for example, measurements should not be taken only prior to drug administration). We also suggest investigators conducting economic evaluations should release their modelling as open data to maximise transparency and further research in the field.

## Conclusion

Overall we conclude that although there has been progress since 2013 [9], there are still only a small number of high quality independent economic evaluation studies in NEN. Most HEEs do not meet published health economic criteria used to assess quality. Clinicians should be cautious when interpreting economic evaluations of high-cost treatments of NEN given the complexities associated with comparisons across heterogeneous trials with confounding of relevant outcomes. Further research with high-quality effectiveness data and rigorous applied health economic analysis is needed.

## Appendix 1: Search strategy

“((((exp “NEUROENDOCRINE TUMOR“/ OR (“neuroendocrine tumour“ OR “neuroendocrine tumor“).ti,ab OR exp “GASTROENTEROPANCREATIC NEUROENDOCRINE TUMOR”/ OR (“gastroenteropancreatic neuroendocrine tumour“).ti,ab OR (“gastroenteropancreatic neuroendocrine tumor“).ti,ab OR exp CARCINOID/ OR (“carcinoid tumor” OR “carcinoid tumour”).ti,ab)

AND

(OCTREOTIDE/ OR ANGIOPEPTIN/ OR “ALPHA INTERFERON”/ OR “ALPHA2B INTERFERON”/ OR EVEROLIMUS/ OR RAPAMYCIN/ OR SUNITINIB/ OR “LUTETIUM 177”/ OR (octreotide OR lanreotide OR interferon-a OR interferon alpha-2b OR everolimus OR sirolimus OR sunitinib OR 177Lu).ti,ab OR CHEMOTHERAPY/ OR RADIOTHERAPY/ OR “TUMOR ABLATION”/ OR RADIOEMBOLIZATION/ OR “ARTIFICIAL EMBOLIZATION”/ OR SURGERY/ OR (“trans arterial chemoembolization”).ti,ab OR TELOTRISTAT/ OR (chemotherapy OR radiotherapy OR ablation OR radioembolisation OR radioembolization OR embolization OR embolization OR surgery OR telotristat).ti,ab))

AND

(HOSPITALIZATION/ OR “HOSPITALIZATION COSTS”/ OR (hospitalisation OR hospitalization).ti,ab OR “COST OF ILLNESS”/ OR (“cost of illness”).ti,ab OR MORTALITY/ OR (mortality).ti,ab OR “AMBULATORY CARE”/ OR (“ambulatory care”).ti,ab OR (“work loss”).ti,ab OR DISABILITY/ OR (disability).ti,ab OR “HEALTH CARE COST”/ OR (“healthcare cost*”).ti,ab OR (“health care cost*”).ti,ab OR (“treatment pattern*”).ti,ab OR “DRUG UTILIZATION”/ OR (“drug utilisation” OR “drug utilization”).ti,ab OR (“unmet needs”).ti,ab OR “QUALITY OF LIFE”/ OR (“quality of life”).ti,ab OR (“health economic analysis”).ti,ab OR “HEALTH ECONOMICS”/ OR “HEALTH CARE UTILIZATION”/ OR (“resource utilisation” OR “resource utilization”).ti,ab OR (“financial toxicity” OR “cost-effectiveness” OR economics).ti,ab OR (“cost of disease” OR “cost of sickness” OR “burden of disease” OR “burden of illness” OR “hospital cost” OR “cost of medical care” OR “cost of drug*” OR “cost of healthcare” OR “cost of health care” OR “cost of treatment” OR “treatment cost” OR “illness cost” OR “health* cost” OR “drug cost” OR “medical care cost” OR “sickness cost” OR “economic burden”).ti,ab OR (“economic impact” OR “health services” OR “drug prescriptions” OR “direct cost” OR “intangible cost” OR “surg* cost” OR payment OR economic OR pharmacoeconomic OR absenteeism OR “illness day” OR “sick day” OR “sick leave” OR “work absen*” OR retirement OR “work day loss” OR “work incapability” OR “work loss” OR “work day loss” OR “work time loss” OR “working incapacity” OR “workers compensation”).ti,ab))

AND

((Registry OR registries).ti,ab OR REGISTER/ OR “OBSERVATIONAL STUDY”/ OR (“observational stud*”).ti,ab OR exp. REVIEW/ OR (review).ti,ab OR “PRACTICE GUIDELINE”/ OR (guideline*).ti,ab OR CONSENSUS/ OR (consensus).ti,ab OR (“meta-analysis” OR “systematic review” OR survey*).ti,ab)) [DT 2010–2019] [English language]”.

## Appendix 2: Critical appraisal

**Table:** Critical appraisal was conducted using the Philips checklist [47]

Author & Year	S1	S2	S3	S4	S5	S6	S7	S8	S9	D1	D2a	D2b	D2c	D3	D4a	D4b	D4c	D4d	C1	C2
Takemoto 2010 (abstract) [58]	Y	N	N	N	N	U	Y	Y	U	N	U	U	NA	N	N	N	N	N	N	N
Soares 2011 (abstract) [54]	Y	Y	N	N	N	U	Y	Y	U	N	U	U	NA	N	Y	N	N	N	N	N
Walczak 2012 (abstract) [61]	Y	Y	N	N	N	U	Y	U	U	N	U	U	U	N	Y	N	N	N	N	N
Marty 2012 [37]	Y	N	N	N	Y	Y	N	NA	NA	N	Y	Y	NA	Y	N	N	N	N	N	N
Johns 2012 (abstract) [26]	Y	Y	N	N	N	U	U	U	U	N	U	U	U	N	U	U	N	U	N	N
[28] (abstract) [28]	Y	Y	N	N	N	U	Y	Y	U	N	U	U	U	N	N	N	N	N	N	N
Casciano 2012 [7]	Y	Y	N	Y	N	N	Y	Y	Y	Y	Y	N	N	Y	N	N	N	Y	N	N
Spolverato 2015 [55]	Y	Y	N	Y	N	Y	Y	N	Y	N	N	N	N	Y	N	N	Y	Y	Y	N
Joish 2018 [27]	Y	Y	N	N	Y	Y	Y	Y	Y	N	Y	N	N	N	N	N	N	Y	N	N
Mujica-Mota 2018 [41]	Y	Y	Y/N	Y	Y	N	Y	Y	Y	Y	Y	U	Y	Y	Y	Y	Y	Y	Y	Y
Chua 2018 [10]	Y	Y	N	Y	Y	N	Y	Y	Y	N	Y	N	Y	Y	Y	Y	N	Y	N	N
Ray 2019 (abstract) [50]	Y	N	N	N	U	U	U	U	NA	N	NA	NA	NA	N	N	N	N	N	N	N

Key: *Y* Yes fulfils criteria, *N* No does not fulfil criteria, *NA* Not applicable
